# Developing an evidence-based program sustainability training curriculum: a group randomized, multi-phase approach

**DOI:** 10.1186/s13012-018-0819-5

**Published:** 2018-09-26

**Authors:** Rebecca Vitale, Timothy Blaine, Elizabeth Zofkie, Sarah Moreland-Russell, Todd Combs, Ross C. Brownson, Doug A. Luke

**Affiliations:** 1 Prevention Research Center in St. Louis, Brown School at Washington University in St. Louis, 1 Brookings Drive, Campus Box 1196, St. Louis, MO 63130 USA; 2 Center for Public Health System Science, Brown School at Washington University in St Louis, St Louis, MO USA; 3 Department of Surgery (Division of Public Health Sciences) and Alvin J. Siteman Cancer Center, Washington University School of Medicine, Washington University in St. Louis, St. Louis, MO USA

**Keywords:** Sustainability, Organizational sustainability, Dissemination and implementation, Tobacco control, Evidence-based practice, Institutionalization, Experiential learning, Action-oriented, Technical assistance

## Abstract

**Background:**

The emergence of dissemination and implementation (D&I) science has driven a rapid increase in studies of how new scientific discoveries are translated and developed into evidence-based programs and policies. However, D&I science has paid much less attention to what happens to programs once they have been implemented. Public health programs can only deliver benefits if they reach maturity and sustain activities over time. In order to achieve the full benefits of significant investment in public health research and program development, there must be an understanding of the factors that relate to sustainability to inform development of tools and trainings to support strategic long-term program sustainability. Tobacco control programs, specifically, vary in their abilities to support and sustain themselves over time. As of 2018, most states still do not meet the CDC-recommended level for funding their TC program, allowing tobacco use to remain the leading cause of preventable disease and death in the USA. The purpose of this study is to empirically develop, test, and disseminate training programs to improve the sustainability of evidence-based state tobacco control programs and thus, tobacco-related health outcomes.

**Methods:**

This paper describes the methods of a group randomized, multi-phase study that evaluates the empirically developed “Program Sustainability Action Planning Training” and technical assistance in US state-level tobacco control programs. Phase 1 includes developing the sustainability action planning training curriculum and technical assistance protocol and developing measures to assess long-term program sustainability. Phase 2 includes a group randomized trial to test the effectiveness of the training and technical assistance in improving sustainability outcomes in 24 state tobacco control programs (12 intervention, 12 comparison). Phase 3 includes the active dissemination of final training curricula materials to a broader public health audience.

**Discussion:**

Empirical evidence has established that program sustainability can improve through training and technical assistance; however, to our knowledge, no evidence-based sustainability training curriculum program exists. Therefore, systematic methods are needed to develop, test, and disseminate a training that improves the sustainability of evidence-based programs.

**Trial registration:**

NCT03598114. Registered 25 July 2018—retrospectively registered.

## Background

Demonstrating the effective implementation of a program is only the first step in influencing the health and wellness of a target population. For a population to experience the benefits of an implemented evidence-based intervention, the intervention must be sustained over time. Program sustainability is a complex process, often fraught with challenges [1–4]. Research consistently indicates that even effectively implemented interventions risk failure when funding, planning, or training ends [5–8]. In fact, it is estimated that up to 40% of programs end within 2 years of losing funding [9]. Failure to sustain an implemented program negatively impacts communities through loss of trust in public health initiatives and waste of valuable resources [10].

As of 2013, 42.1 million—or one in five—adults in the USA smoke, leading to an estimated 480,000 deaths per year due to tobacco use [11]. According to the Centers for Disease Control and Prevention (CDC), if smoking continues at its current rate, more than five million of today’s youth will die prematurely from smoking-related disease and the economic cost will rise to over $300 billion per year [11]. All the while, tobacco use has long been identified as a major *preventable* cause of death and disease [12]. Given the burden of tobacco use, it is imperative that high-quality, state tobacco control programs exist and are sustained.

The goal of this study is to increase capacity for sustainability among evidence-based tobacco control programs. Although all 50 states have implemented evidence-based tobacco control programs and policies, each program varies in its ability to support and sustain itself over time. Tobacco control funding directly correlates with US adult smoking rates in that states with more funding have lower smoking rates [12]. Between 1985 and 2003, adult smoking prevalence declined from 29.5 to 18.6%, due to increases in tobacco control funding [12]. Farrelly et al. estimated if, starting in 1995, all states funded their TC programs at the optimal levels recommended by the CDC, there would have been 2.2 million to 7.1 million fewer smokers by 2003 [12]. Given the established evidence for state tobacco control program and the work left to do in the field, it is essential to sustain the state tobacco control programs, to both improve quality of life and reduce the massive healthcare risks incurred by smoking-related illness [12].

This study defines sustainability as the existence of adaptive structures and processes that enable a program to effectively implement and institutionalize evidence-based policies and activities over time [1]. Empirical evidence has established that program sustainability can be improved through in-person, hands-on, action-oriented training and technical assistance [13–16]. Research also highlights the importance of creating an action plan to move sustainability progress forward [17]. Sustainability planning predicts program survival and post-launch funding [17]; however, to date, no evidence-based sustainability training curriculum exists. While there is a growing body of research on aspects affecting sustainability [2–6, 18], little has been done to translate the components of program sustainability capacity into practical guides and tools for practitioner utilization. Thus, this study aims to develop and validate the first evidence-based program sustainability action planning model and training curriculum and ultimately disseminating training curricula materials to broader public health audiences.

## Methods/design

### Study design

This study, funded by the National Cancer Institute, seeks to develop and evaluate the effectiveness of a program sustainability action planning training curriculum in increasing the capacity for sustainability among evidence-based tobacco control programs. The study will be directed by a multidisciplinary team of researchers and practitioners with expertise in: program sustainability assessment, tobacco control program and policy evaluation, planning and training, and dissemination and implementation science. This study team consists of researchers from the Prevention Research Center (PRC) at Washington University in St. Louis with collaboration from the Center for Public Health Systems Science (CPHSS) at Washington University in St. Louis and CDC’s Office on Smoking and Health (OSH). The Institutional Review Board of Washington University in St. Louis and the Protocol Review and Monitoring Committee approved all study procedures.

**Fig. 1:**
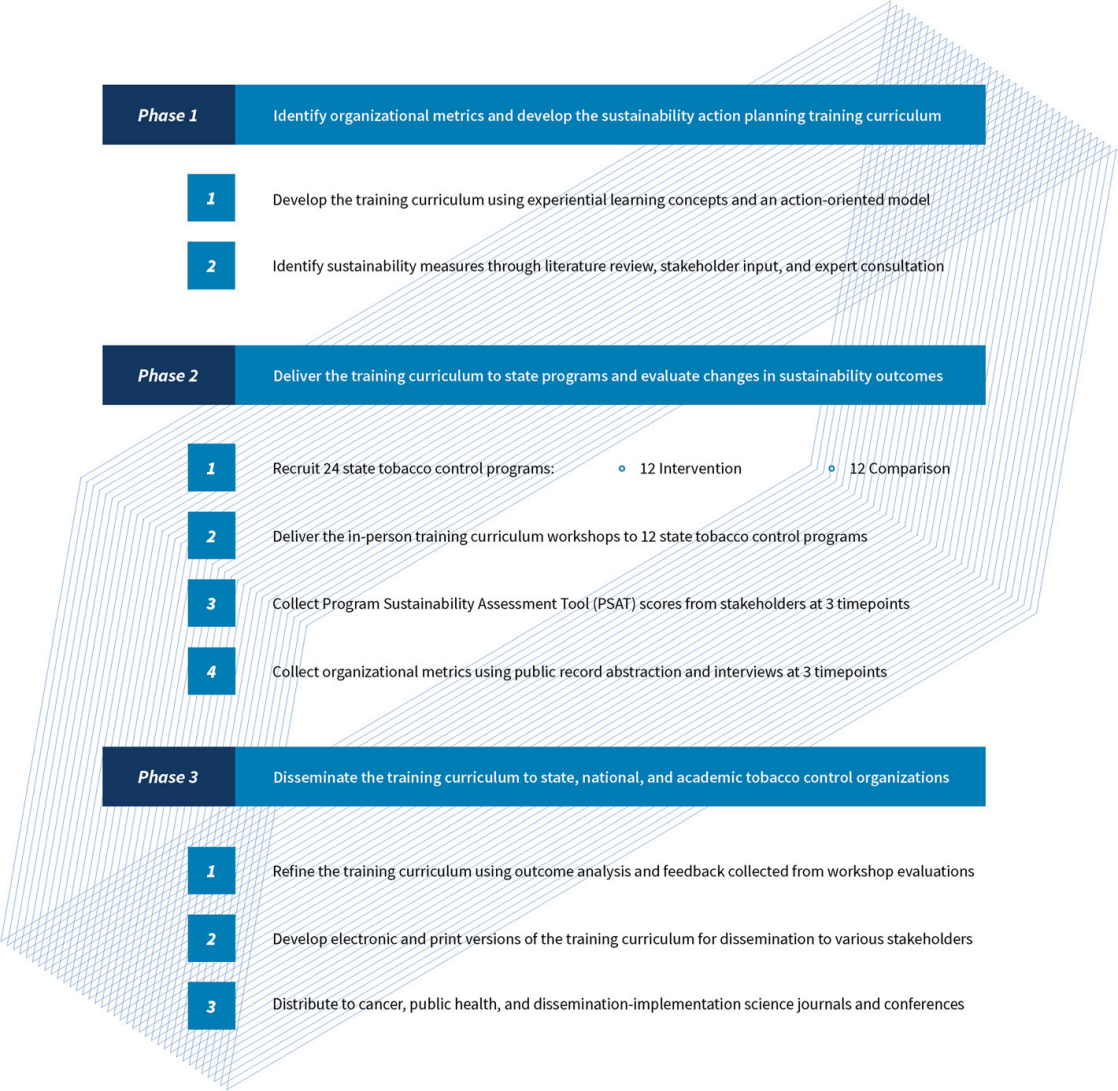
Study schema

The study involves three interconnected phases. Phase 1 consists of the development of a sustainability action planning training curriculum including the creation of an interactive workbook and design of a training workshop. Additionally, Phase 1 will focus on the development of surveys, interviews, and record abstraction protocols to collect programmatic, organizational, community-level, and funding data associated with program sustainability and institutionalization. Phase 2 is a group randomized, mixed methods study designed to assess the training curriculum’s effectiveness in increasing the capacity for sustainability among 24 statewide tobacco control programs. This involves two parallel study arms with 12 intervention states and 12 pair-matched comparison states. States will be paired together according to an analysis of programmatic factors, then randomly selected into intervention or comparison groups. Those programs placed in the intervention condition will receive numerous training strategies, including in-person sustainability action planning training workshops, ongoing technical assistance, and periodic communications regarding project progress as well as recent program sustainability and tobacco control news. State programs participating in the comparison condition will also be encouraged to create a sustainability plan, but they will receive neither training workshops nor technical assistance. Phase 3 will focus on both refining the training curriculum based on evaluation following implementation and actively disseminating all training materials to broader public health audiences. Figure 1 depicts a visual of the study schema, describing the components of each study phase. Some aspects of phase 1 have been completed while phase 2 planning and recruitment is underway.

### Conceptual model

**Fig. 2:**
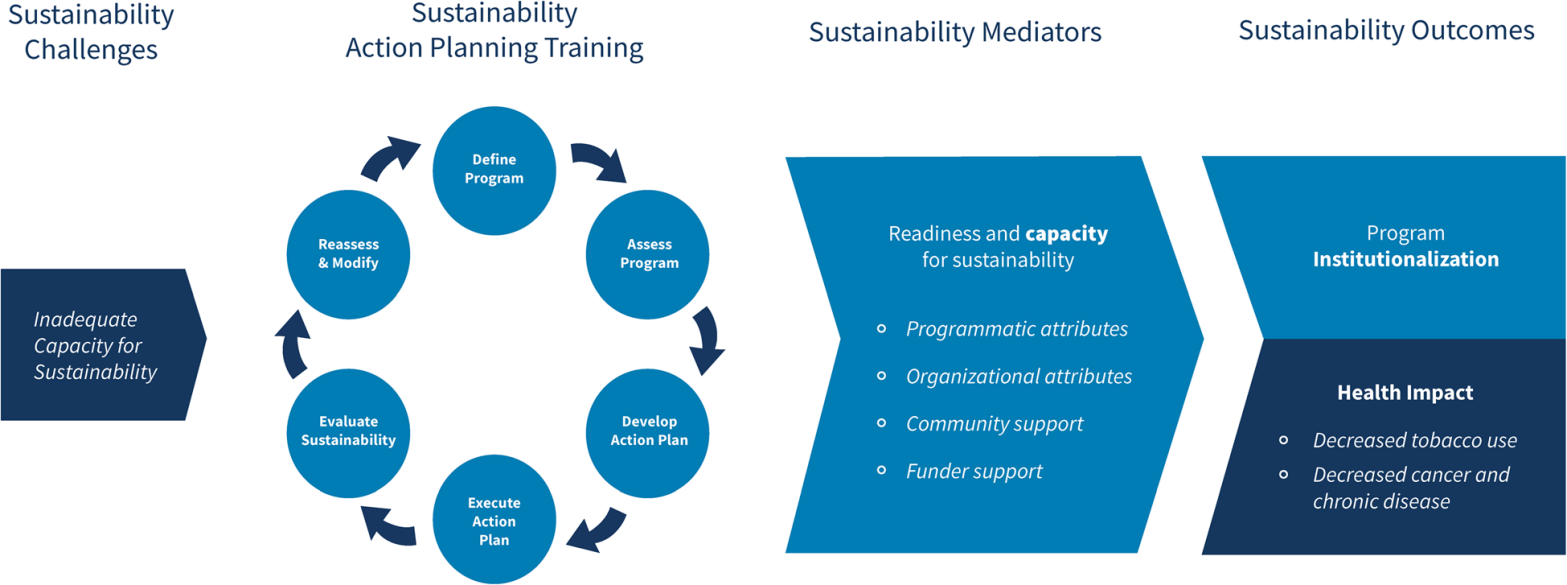
Sustainability theory of change conceptual model

The conceptual model for the study is driven by the theory of change [13, 19–21]. Figure 2 illustrates the theory of change conceptual model for this project, adopted from Johnson’s conceptual model of sustainability planning for substance abuse service coalitions [13, 21]. The model outlines a set of causal factors that help in evaluating the effectiveness of the program sustainability action planning model and training curriculum in increasing capacity for sustainability. Sustainability planning played out through a six-step process can directly affect sustainability readiness and capacity for sustainability as defined by enhancement of programmatic and organizational attributes, community stakeholders, and funder support. Increased readiness and capacity for sustainability will mediate the effect of the sustainability action planning training on sustainability success in the form of institutionalization. The study defines institutionalization as the continuous integration of the program into normal operations of the organizational system. The resulting health impact follows the sustainment of the evidence-based program over time and is regarded as a decrease in incidence of tobacco use, cancer, and chronic disease.

### Study audience

The target audience for this study includes statewide tobacco control programs and their stakeholders, drawn from diverse organizations such as state and local health departments, community-based organizations, universities, and policymakers. All participants will be non-institutionalized adults ages 18 and over. The only exclusionary criterion is that participants must be involved in their state’s tobacco control program and must be selected by their state program manager as a stakeholder. Historically, tobacco control programs endure constant changes in funding and political support [12]. Similarly, sustainability is a pressing issue in the public health sector as a whole [3, 8]. Finite funding opportunities propel many public health programs, so changes in funding often curtail hard-earned advancements. Therefore, phase 3 will focus on wider dissemination to a broader audience. To this end, measures and evaluation criteria were designed for future adaptation to any public health program or initiative.

### Phase 1: Development of curriculum and measures

#### Training curriculum development

**Fig. 3:**
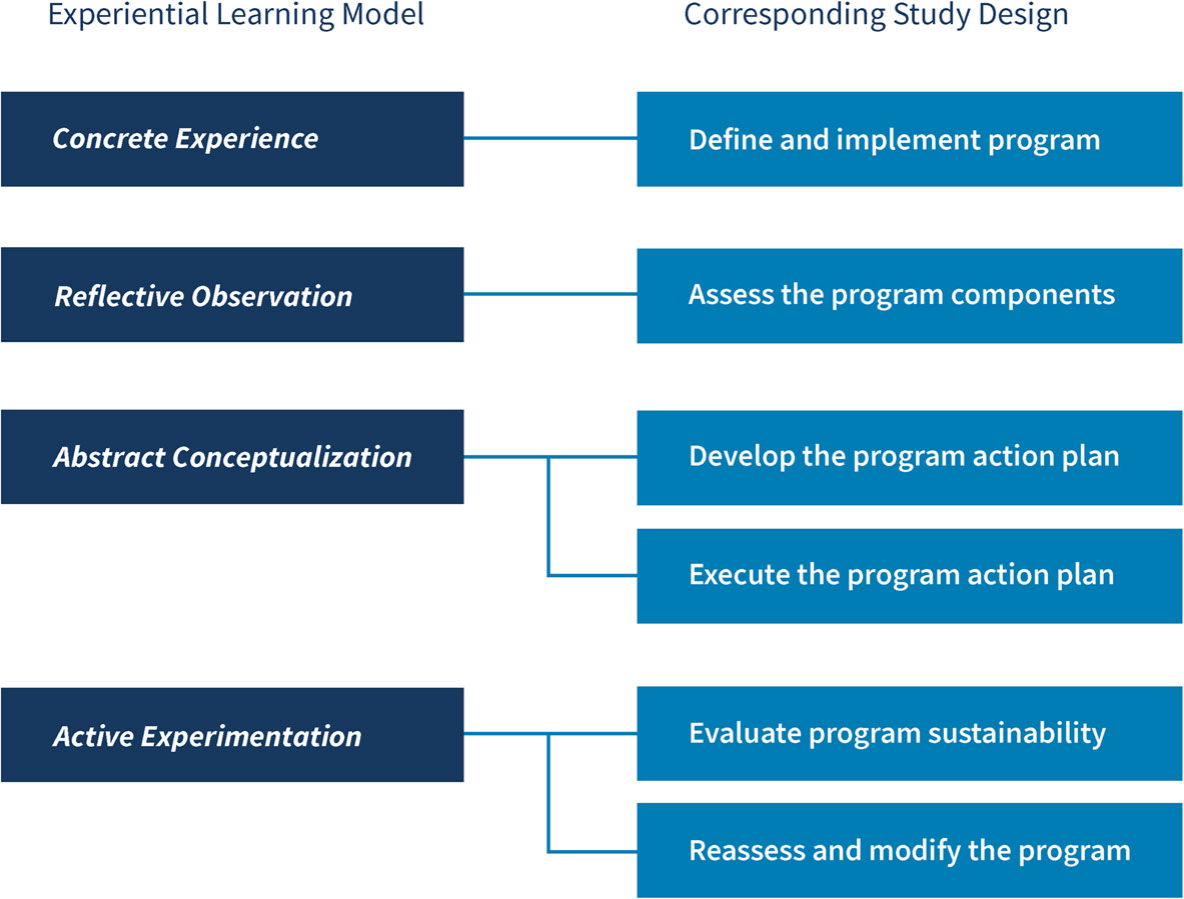
Kolb’s learning theory model aligned with the study design

The study team will develop and deliver a series of in-person training workshops to educate stakeholders about program sustainability and facilitate their completion of a sustainability action plan. The study team will develop a workbook for each participant to use with the training curriculum. The workshop will elaborate upon previous trainings conducted by CPHSS and empirical recommendations [22–28] to incorporate Kolb’s four-step experiential learning model [22]. Experiential learning is a process comprised of four elements: concrete experience, reflective observation, abstract conceptualization, and active experimentation [10]. Developed with this in mind, the theory and the model of this study emphasize active engagement. The core principles of active engagement include defining and implementing the program (concrete experience), assessing the program (reflective observation), developing and executing the action plan (abstract conceptualization), evaluating sustainability, and reassessing and modifying (active experimentation). Fig. 3 illustrates the alignment of these principles with the study design. Providing balanced learning experiences and incorporating virtual, remote, and hands-on sessions and reflection—as the current study will do—have shown to lead to deeper learning and maximum information retention [22, 29].

Complementary to these core principles, the study team set four goals to guide the development of the training curriculum workbook: (1) to offer an interactive guide for participants to return to as a sustainability planning resource; (2) to spark engaging, transparent, and collaborative discussions; (3) to communicate with a range of community-oriented organizations; and (4) to remain readable across print and digital formats. These goals, in combination with the experiential learning framework, will motivate the study team to design interactive activities throughout, including fill-in visualizations, journaling segments, and discussion prompts. In each section, the audience will apply educational material to the context of their program. The training workbook will mirror the format of the action planning workshop, and stakeholders will reference the workbook throughout the course of the training. In the following years, the workbook will serve as a resource encouraging proactive and accountable use of the action plan and accompanying sustainability planning resources.

Technical assistance after the training will promote continuation of the action plan and provide troubleshooting for the intervention states. This is an essential step in the action planning process to help build each state program’s capacity for quality implementation of the action plan. Following each intervention, flexible and proactive technical assistance with clearly defined responsibilities and tailored messaging will strengthen communication between providers and recipients [30]. Through consultations with the advisory board and data from an ongoing literature review, the study team elected to issue quarterly feedback, provide on-call troubleshooting assistance from academic experts, allow for state programs in the intervention condition to share insights, and send custom activities and tools in the years following training.

#### Data collection and management

Following recommendations from the advisory board and tobacco control experts, the study team will collect data primarily using record abstraction. This will help to alleviate the burden of an intensive, all-encompassing survey or interview. However, because it may be infeasible to collect all data points through program records, the study team has also developed a key informant interview tool to collect remaining information. The interviews will be conducted via a 15–20-min phone interview with state program managers or any other qualified surrogate. Additionally, all answers will be recorded, transcribed, and reviewed for completeness and accuracy. An online Qualtrics survey will be developed with identical questions for the convenience of state program managers that prefer not to complete a phone interview. Each interview or survey will be coded by assigned team members and checked for inter-rater reliability.

Much of the record data for this study will come from annual state-level reports to the Office of Smoking and Health at the CDC. These reports address fulfillment criteria for the DP15-1509 funding announcement and describe the infrastructure, personnel, and activities of state tobacco control programs in detail. These funding announcements are a requirement of state programs, set by the CDC, to complete yearly reports of progress, goals, and challenges in order to receive federal funding. The study team has strategically timed this project to coincide with the annual release of these reports. In addition to the CDC reports, other data will be collected via secondary data sources, including the American Lung Association’s annual *State of Tobacco Control* report [31] and the annual *Healthy Americans* report issued by Trust for America’s Health [32].

Phase 1 also includes production of a codebook to organize and explain the programmatic and organizational data collected, as well as a record abstraction protocol. The project codebook will align each data point within four key areas (programmatic attributes, organizational attributes, community-level factors, and funder support) [33].

#### Measures

The program sustainability framework was developed from previous research completed by the CPHSS team through an extensive literature review, concept mapping, and expert input [1, 33]. The framework defines the internal and external factors operationalized into eight domains that affect an organization’s capacity for sustainability including environmental support, funding stability, partnerships, organizational capacity, program evaluation, program adaptation, communications, and strategic planning. The Program Sustainability Assessment Tool (PSAT) consists of 40, 7-point Likert-scale items organized into the eight domains of the program sustainability framework.

Table 1 describes these variables, their type, their method of collection, and their basis in previous program sustainability research. The presence or absence of the action planning training curriculum is both the intervention and independent variable for this study. Program sustainability is the study’s main outcome measure and will be measured by four empirically established indicators of institutionalization: (1) the anchoring of a program in law, regulation, or other organizational rules; (2) the inclusion of a program in the regular organizational budget; (3) the percentage of an organizational budget allotted to the program; and (4) the percentage of CDC-recommended tobacco control funding level spent [8, 33]. The PSAT scores will be evaluated as an outcome against these four conventional measures.

**Table 1:** Sample measures

Variable description	Variable type	Collection method	Collection source	Literature	Description
Program Sustainability Assessment Tool (PSAT) aggregate scores	Dependent	PSAT	State program stakeholders	[1, 14, 36]	A survey tool composed of 40 7-point Likert scale questions. Survey items solicit perceptions of sustainability across eight conceptual domains.
Whether the state tobacco control program is established or continued through state statute or regulation, and/or if the program is stated in organizational doctrine as a permanent component of the auspice organization	Dependent	Key informant interview	State program manager	[33]	A large-scale study found that the inclusion of a program in law or other organizational rules significantly predicted the continuation of that program. Of the observed programs, “50.7% were anchored in law.”
Whether the tobacco control program is included as part of the regular state budget as an ongoing budgetary line item	Dependent	Key informant interview	State program manager	[33]	Study of program sustainability observed that 59% of its continued programs were permanent budgetary items, with 75% of programs having some budgetary inclusion also continuing.
Percentage of organizational budget allotted to tobacco control program (e.g., the amount allotted from the budget of the state health department)	Dependent	Key informant interview	State program manager	[33]	In addition to inclusion within the budget, the amount of organizational budget spent on the program was found to predict sustainability.
The percentage of the CDC-recommended funding level for tobacco control programs actually spent in a given year	Dependent	Record abstraction	American Lung Association: *State of Tobacco Control*; Trust for America’s Health: *Healthy Americans* report	[33]	The CDC-recommended funding level represents an empirically determined optimal level of funding. The percentage of this funding level actually spent evaluates the support given to tobacco control programs by state organizations.
Program manager’s years of experience in tobacco control	Mediator	Key informant interview	State program manager	[38]	The capacity and duration of a capable leader were identified as one of the top indicators of sustained implementation among programs surveyed.
Development status of the tobacco control program’s plan for sustainability	Mediator	Record abstraction	CDC annual reports: benchmarks and performance measures	[47]	The CDC recommends “planning for program sustainability at the beginning rather than at the end of a funding cycle.” Details about the time and status of this plan’s development will be pertinent to the implemented sustainability it describes.
Existence of a plan to continue funding in light of sudden decreases in funding from existing sources	Mediator	Key informant interview	State program manager	[43]	There exists a strong positive correlation between a program’s diversity of funding resources and its likelihood to continue operation.
Number and type of staff positions supported by the tobacco control program	Mediator	Key informant interview	State program manager	[41]	By increasing staffing positions and hours, programs transcend basic operations and become proactive in applying for new sources of support. The types of positions covered by the program echo in CDC’s Best Practices recommendations

#### Mediators

As aforementioned, increased readiness and capacity for sustainability will mediate the effect of the workshop on sustainability success or institutionalization. Each mediating variable contributing to sustainability readiness consists of several factors [33]. Programmatic attributes include amount of funding allotted, diversity of funding sources, staff size and commitment, leadership abilities and commitment, and the overall scope and mission of the program [33]. Because program manager turnover may result in changes to planning, the study team will create a variable to account for the potential influence of a change in leadership. Associated organizational attributes include the organization’s size, the organizations leadership’s perceptions of the program, and the involvement of organizational leadership and staff in the program [33]. Community-level factors include the number of stakeholders involved in the program [33] and the presence of a coalition. Finally, funder support concerns the percentage of the program’s budget covered by external resources [13, 33–35].

Through a systematic literature review, the study team identified indicators of program sustainability that further characterized each key metric [33, 34, 36–46]. In this review, objective measurements of the program became apparent (e.g., manager tenure, staff positions [41], turnover [33, 47] training opportunities [42], and the frequency and magnitude of funding changes [46]). In the community, the presence and operations of a coalition took on particular emphasis as a predictor of sustainability and institutionalization [33, 39]. After the review, the study team mapped each candidate variable to one of four categories (programmatic attributes, organizational attributes, community-level factors, and funder support). Where applicable, the study team contextualized the variable for tobacco control practice. For example, for a standard measure of coalitions, the study team consulted the CDC’s *Best Practices for Comprehensive Tobacco Control Programs* [47] to detail types of organizations tobacco control programs partner with, e.g., retail tobacco organizations, healthcare providers, voluntary health organizations, educational organizations, etc. This tactic led to the discovery of additional coalitional measures, such as the existence of a memorandum of understanding (MOU) between tobacco control programs and universities [45].

While in initial development, these indicators were subject to several rounds of review by academic investigators on the team. In April of 2018, the study team assembled a panel of expert academics and practitioners to deliberate the list of candidate variables and discuss a strategy for collecting the data. This produced a final list of mediating variables. The study team then aligned these indicators with items annually reported by state tobacco control programs to the Office on Smoking and Health at the CDC.

### Phase 2: Implementation with state tobacco control programs

#### Overview

Phase 2 will include a longitudinal group randomized-effectiveness trial to determine differences in organizational and programmatic measures and program sustainability assessment scores between intervention and comparison groups. There will be three staggered recruitment stages with 12 intervention states and 12 paired comparison states. Participating intervention state programs will work closely with the study team to create and carry out their action plan through involvement in technical assistance activities. Although comparison states will receive neither the training curriculum nor technical assistance, they will be given resources to independently construct and carry out a sustainability action plan. Evaluation measures will occur at three time points (baseline, year one, and year two) and will incorporate PSAT scores, program record abstraction, and key informant interview data.

#### State selection and recruitment

State tobacco control program selection and recruitment are based on four criteria: policy progress, resources, need, and previous participation in sustainability training. These characteristics were used to stratify the states when they were assigned to each condition (intervention or comparison). Stratification was important to help ensure the comparability of the grouped states.

**Fig. 4:**
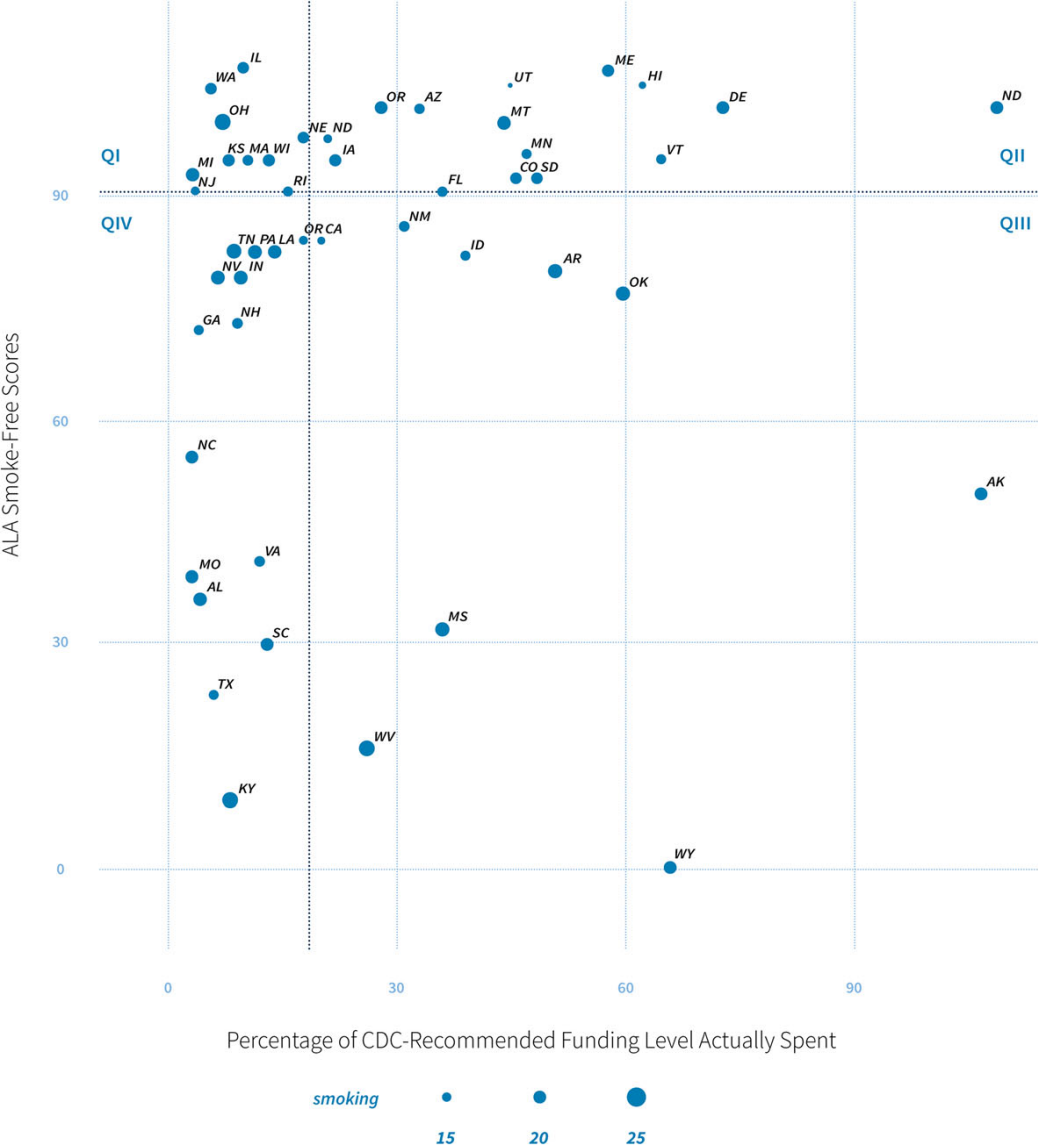
State selection matrix

Tobacco control policy progress is operationalized as the American Lung Association’s (ALA) smoke-free score for each state [31]. This analysis uses national adult smoking rates within each state. Funding is measured as the actual amount of money spent on tobacco control as a percentage of the amount of spending recommended by the CDC. Figure 4 depicts how each of the 50 states in the USA was divided into quadrants based on their ALA scores and CDC-recommended funding. The sizes of the points for each state represent adult smoking rates. The 16 states in quadrant I (top-right) include states that scored well for smoke-free policy although their tobacco control programs are funded at relatively low amounts. Moving counter-clockwise around the plot, the ten states in quadrant II also have high ALA scores yet have higher funding for programs. The nine states in Quadrant III and 15 states in Quadrant IV have the lowest ALA scores with higher and lower funding rates, respectively. All quadrants exhibit variation in adult smoking rates.

The goal is to select three states from each quadrant per group, for a total of six per quadrant, with varying levels of need to build comparable and diverse groups. For example, from each quadrant, two states were selected with relatively low-smoking rates, two from the middle, and two with high-smoking rates. All states selected have not previously participated in sustainability action planning training. Based on a priori analysis, the study team estimated a range of effect size using data from a natural experiment. The data consists of pre- and post-PSAT averages from previous work with five tobacco control programs that received action planning training and five programs that did not. To determine effect size, *t* tests of differences-in-means were conducted for the two groups using the changes in PSAT scores between pre- and post-intervention data. The standardized differences in *t* statistics yielded an effect size of 1.25, which is a large effect size according to Cohen’s criteria [48]. Using the R statistical environment with packages from Del Re [49] and Champely [50], the effect size with power = 0.8 and α = 0.05 projects the necessary sample size to be approximately nine (programs) per group. Since these calculations are based on natural experiments, the study team chose to conservatively propose a sample size larger than calculated due to changes in recommended action planning. This brought the sample size to 12 per group. This sample is sufficient to measure the main goal of the study, which is to discern whether the training and accompanying PSAT tool significantly increase institutionalization of state tobacco control programs [49–51].

State selection and enrollment will be staggered by three pairs at a time during the first two stages of recruitment and six pairs during the final stage over years two and three. The study manager will invite each of the chosen states’ program managers to participate in the study. The program managers will then select between five and 12 key program stakeholders or partners within their state to participate. After enrollment and baseline data collection, the paired states will be randomly assigned into intervention or comparison groups via simple random concealed allocation performed by the statistician. If any program declines to participate, a replacement will be selected from the same quadrant by identifying the state with the closest ALA scores and funding levels.

#### Intervention delivery

In each intervention state, a primary group of tobacco-control stakeholders and study-team members will collaborate to identify and define programmatic aspects of the statewide initiative, including program mission and vision, target populations, strengths and weaknesses, and key partners within and outside of the program. Additionally, these states will participate in sustainability action planning that responds to an in-depth discussion of aggregate PSAT results by addressing particular framework domains. The workshops will consist of a two-day presentation at the state health department. On the first day, facilitators will guide states through defining their tobacco control program and reflecting on their PSAT scores. Participants will consult their scores to frame appraisals and activities addressing strengths and weaknesses of their program and guide the creation of their sustainability action plan. The second day consists of prompts related to creating specific, measurable, attainable, realistic, and time-sensitive (SMART) objectives; activities for participants to link areas of their program and evidence-based topics in sustainability; templates for determining actions and a timeframe; and guidance on creating clear outcomes. The creation of the sustainability action plan is the focal point of the training. The training curriculum will emphasize specific and concrete plans to increase the likelihood that plans are implemented [23, 25]. Intervention strategies will promote hands-on, active learning as described by Kolb’s experiential learning theory. From the end of the training workshop until the end of the study period, the intervention group will participate in ongoing technical assistance with the study team as described above.

#### Evaluation for phase 2

Evaluation measures will be taken at three time points: pre-intervention (baseline) and at 1 and 2 years post-intervention. The study team expects that with the presence of an in-person, customized sustainability training curriculum will correspond with increases in established programmatic and organizational metrics of sustainability. Several process measures will be collected to assess the effectiveness of the training and support delivered. All measures will be collected via survey at the completion of each training component or tracked by review of programmatic records. Quantitative data collected will be analyzed using descriptive statistics while qualitative analysis of programmatic records will be conducted following the PSAT and program record abstraction procedures. This analysis will include dosage delivered, dosage received, and participant reactions. Dosage delivered is the number of hours of training and technical assistance provided to each state. Dosage received will be measured as the extent and frequency that state programs utilize their action plans and project resources and materials. All data will be collected via an evaluation form filled out by state program managers. First, state programs will indicate whether or not an action plan has been created and implemented (e.g., yes, no). To describe frequency of use of their action plan, state programs will provide estimates of how often program personnel discuss and implement the action plan (e.g., hours per month). To describe the extent of use, state programs’ evaluations will report S.M.A.R.T. objectives their stakeholder group determined at either training workshops (experimental condition) or independent meetings (comparison condition) as well as the specific steps this group outlined to achieve those objectives. At each time point, programs will note which steps they have completed under each S.M.A.R.T. objective. The resulting ratio of complete action plan steps to incomplete action plan steps will represent dosage received. This ratio measurement will allow comparisons between state programs with action plans of different lengths. Program managers will also indicate whether or not their state program has utilized project resources and materials (e.g., yes, no). They will also describe their frequency of use of these resources and materials (e.g., daily, several times/week, several times/month, once/month, less than once/month).

In addition, participant reactions will be measured by the extent to which they felt that the objectives of the training and technical assistance were achieved. They will also be asked to report levels of satisfaction with, and their perceptions of the usefulness of, the training and technical assistance provided. This qualitative data will be collected through a post-training evaluation and will inform the study team of any limitations of the format and content as well as ways in which the training can be improved in the future. This feedback will assist the study team to modify the curriculum for later dissemination.

#### Data analysis

Descriptive statistics, including frequencies and measure of central tendency and dispersion, will be calculated for both the intervention and comparison groups at each of the three data collection time points to assess baseline averages and changes. To incorporate the influence of each state’s distinctive characteristics, longitudinal regression analysis will be used to model the outcome for each of the dependent variables. This analytic model is described by the following equation:

$$ {Y}_{it}={\beta}_0+{\beta}_1{PSAT}_{it=0}+{\beta}_2{G}_i+{\beta}_3{D}_{it}+{\beta}_4\left({G}_i\ast {D}_{it}\right)+\dots +{\beta}_k{X}_{k, it}+{u}_{it} $$

where *Y* is the outcome variable and *i* = state and *t* = time point (1 or 2); *PSAT*_*it* = 0_ is the baseline PSAT score, *G*_*i*_ signifies the group (intervention or comparison), *D*_*it*_ signifies dosage at time *t*, while (G_*it*_ * D_*it*_) represents an interaction between the group and dose terms; *X*_*k*,*it*_ represents a vector of programmatic, organizational, community, and funder variables, and *u*_*it*_ is the error. This analysis will ultimately be used to test the hypothesis that the impact of the training is nonzero and positive.

A multivariate approach accounts for the numerous influences found in programmatic, organizational, community, and funder attributes. Additionally, data collection at different time points allows for measuring changes in these variables, and the influence these changes have on institutionalization outcomes. The level of adoption and implementation of action plans both from the training (intervention states) and from other initiatives (comparison states) will vary. This variance in dosage reception and delivery will be acknowledged through multivariate longitudinal analysis.

### Phase 3: Final dissemination strategies

The results of this study will be used in many different ways. Potential final dissemination strategies include:

1. Electronic versions of final workbook and curriculum materials;
2. Instructional videos to support independent action planning work;
3. Standardized reporting forms in partnership with the CDC-OSH to support ongoing data collection and fidelity to the training model;
4. Technical assistance worksheets and activities posted on *www.sustaintool.org*;
5. Webinars;
6. Findings about the impact of the program sustainability action planning model and training curriculum on programmatic and organizational outcomes in high-visibility cancer, public health, and dissemination and implementation science journals and conferences;
7. All lessons learned from the training program and a final curriculum product will be systematically disseminated to diverse audiences, since the methods and approaches are generalizable and adaptable to any public health initiative.

These final dissemination strategies will be provided to both intervention and comparison state tobacco control programs included in the study following its completion. Additionally, the materials will be generalizable and available for any public health program seeking to build capacity for sustainability.

#### Study status

The study is currently in progress. The study team has completed certain phase 1 activities, including an extensive literature review, determination of key metrics and variables, and development of a program record abstraction protocol. The final development of the action planning workbook and other training materials is still ongoing and close to completion. Phase 2 of the study has begun. Recruitment and program record abstraction are in progress for the first round of three paired states.

## Discussion

This study provides a potentially innovative approach to increase the sustainability capacity of tobacco control programs in a number of ways. First, this study will provide the first ever evidence-based program sustainability action planning model and training curriculum. By establishing this evidence-based method for action planning and technical assistance related to sustainability, the study team is supporting state tobacco control programs and other evidence-based public health initiatives to sustain their positive impact, despite tumultuous funding climates. Additionally, there are currently no available resources specifically designed to assist state tobacco control programs in meeting national requirements to “develop a sustainability plan” or “provide measures of execution activities as outlined in the plan” [34]. The development and broad dissemination of this training will assist states to fulfill these requirements and establish their capacity to continue sustainability planning over time. This study will also provide more clarity on the conditions that contribute to sustainability of evidence-based interventions, building on existing literature focusing on effectiveness testing and early-phase implementation.

### Limitations

The study has a few limitations. The main limitation is the possibility of state drop-out. Replacing participating programs will be difficult due to the longitudinal nature of the study*.* The study team hopes to combat this by offering incentives to stay in the study, including use of action planning resources and access to the final dissemination products.

Staff turnover in state tobacco control programs is also a notable concern. Discussions with tobacco control consultants have described the seemingly unavoidable reality of key stakeholder turnover in states. Turnover is likely due to many factors, including lack of funding. This is why this study is particularly timely, as there is potential to acknowledge these hardships and create plans to improve. The study team, however, will monitor staff turnover and manage it by staying in close contact with the state program managers and OSH project officers.

Finally, another important limitation to note is the lack of empirical literature related to technical assistance best practices, particularly within tobacco control programs. This makes it difficult to establish a uniform, evidence-based technical assistance protocol to apply to all states. However, through past experience of the team working with state programs and discussions with tobacco control consultants and a formative systematic literature review, the study team concluded that more flexible approaches for technical assistance may be the most efficient way to assist states, considering that every state is unique. Thus, the team will have technical assistance guidelines but will employ more tailored approaches for each state, based on need. The team hopes to gain insight on technical assistance best practices and describe lessons learned in the dissemination products, building on the small body of knowledge that exists for this topic area.

### Conclusion

This study has the potential to improve public health programs by introducing a rigorous evaluation for program sustainability and creating tools to improve sustainability over time. The benefits of program sustainability will not only benefit the state programs themselves, but also the health of state populations through the continuation of tobacco control initiatives shown to decrease tobacco-related disability and death. This study’s findings will contribute to the growing body of knowledge on how to mature and sustain activities over time, thereby achieving the full benefit of significant public health investment.

## Abbreviations

ALA: American Lung Association
CDC: Centers for Disease Control and Prevention
CPHSS: Center for Public Health Systems Science
D&I: Dissemination and implementation
MOU: Memorandum of understanding
OSH: CDC’s Office of Smoking and Health
PRC: Prevention Research Center
PSAT: Program Sustainability Assessment Tool
SAS: Statistical Analysis System
SMART: Specific, measurable, attainable, realistic, time-sensitive
TC: Tobacco control
